# The association between family history of prostate cancer and development of prostate cancer among Korean population: A prospective cohort study using KoGES data

**DOI:** 10.1097/MD.0000000000024757

**Published:** 2021-02-19

**Authors:** Hyo Geun Choi, Woo Jin Bang, Jung Ki Jo, Cheol Young Oh, Myungsun Shim, Jin Seon Cho

**Affiliations:** a Department of Otorhinolaryngology-Head & Neck Surgery; b Hallym Data Science Laboratory; c Department of Urology, Hallym University College of Medicine, Anyang; d Department of Urology, Hanyang University College of Medicine, Seoul, Korea.

**Keywords:** cohort studies, family, history, prostatic neoplasms

## Abstract

This study aimed to assess the impact of family history (FH) on prostate cancer (PCa) development among a general Korean population. We conducted a prospective cohort study based on the registry records of 211,789 participants in the database of the Korean Genome and Epidemiology Study from 2001 to 2013. A total of 69,693 men with appropriate records were evaluated by being categorizing into 2 groups; a PCa group (100) and control group (69,593). FH of PCa was also categorized as FH of total, father, or brother. Odds ratios (ORs) of PCa development were calculated by using stratified logistic regression models. The adjusted OR of PCa history of father was 27.7 (95% confidence interval [CI] = 9.7–79.2, *P* < .001) in PCa patients compared to control, and that of PCa history of brother was 15.8 (95% CI = 3.6–69.6, *P* < .001). Among the adjusted variables, age (OR, 1.17; 95% CI, 1.14–1.21; *P* < .001), and hyperlipidemia (OR, 2.25; CI, 1.32–3.84; *P* = .003) were also identified as significant predictors of PCa development. There was no difference in the impact of FH on PCa development between different age groups at PCa diagnosis (<60 vs ≥60 years). To our knowledge, this study represents the first prospective cohort study based on the registry data of a Korean population showing the significance of FH on PCa development. Additionally, the effect of FH on the early onset of PCa has not been confirmed in our analysis.

## 1 Introduction

It is well known that there is a risk of over-diagnosing and over-treating prostate cancer (PCa) in a significant number of cases, and performing prostate-specific antigen (PSA)-based screening in men to reduce cancer-specific mortality poses many questions. Although the European Randomized Study of Screening for Prostate Cancer (ERSPC) showed a 20% reduction in PCa-specific mortality by PSA screening, it also demonstrated a significant degree of overdiagnosis.^[1]^ Therefore, a thorough stratification of the risk is important to provide the basis for clinical counseling and recommendations for screening to avoid overdiagnosis.^[2]^

A family history (FH) of PCa is a well-established risk factor for the disease^[3]^ and thus can be useful in conducting consultations for screening and diagnosis of PCa. Previous studies have consistently shown a 2- to 4-fold increased risk in sons and brothers of PCa patients.^[4,5]^ In particular, a large twin study showed that an FH of PCa increases the likelihood of developing PCa in the future, especially in men who have first-degree relatives with PCa.^[6]^ There have been several other studies demonstrating the risk of PCa and FH using large-scale databases.^[7,8]^ However, many studies using registry data were based on records collected from a population register before or at the beginning of the PSA-screening era and, therefore, do not accurately reflect the effects of PSA screening. Furthermore, most of the findings on this topic have been based on Western data and may not exactly apply to an Asian population. In particular, since the incidence of PCa is known to be significantly lower compared to that of Western countries, it may have different characteristics from Western data.^[9,10]^ Indeed, previous literature has shown contradictory results on the impact of FH on the risk of PCa among Asian men.^[11,12]^

In this study, we used data from a large prospective cohort, established after the PSA-screening era had begun, from the Korean government (National Research Institute of Health, Centers for Disease Control and Prevention and the Ministry of Health and Welfare, Korea) to estimate the effect of FH of first-degree relatives on the risk of developing PCa among a general population in Korea. We also analyzed the association between FH and PCa development according to age of onset of the disease in PCa patients in the cohort.

## 2 Methods

### 2.1 Study population and data collection

This cohort study relied on prospectively collected data from The Korean Genome and Epidemiology Study (KoGES) from 2001 through 2013, which consists of community dwellers and participants recruited from the national health examinee registry, aged greater than, or equal to 40 years at baseline. A detailed description of these data was provided in the previous study.^[13]^ Among the multiple population-based cohorts in KoGES database, 2 cohorts which includes KoGES_Ansan and Ansung study and KoGES_health examinee (HEXA) were used in this study. The ethics committee of Hallym University (2019-02-020) approved the use of these data. The institutional review board waived the requirement for written informed consent.

### 2.2 Participant selection

Among 211,798 participants, we excluded women (136,840), participants who lacked records on FH of PCa (n = 4,758), and body mass index (BMI; n = 417); therefore, a total of 69,693 men were evaluated. They were divided into 2 groups; a PCa group (100) and control group (69,593).

### 2.3 Survey

Trained interviewers asked participants about their previous PCa diagnosis, onset of age, and their FH of PCa. Anthropometric and clinical measurements were obtained. Age at registry participation was recorded. Income was categorized into 4 groups by monthly income of household:

- no information,
- lowest (< $1,500);
- middle (≥ $1,500 – < $3,000); and
- highest (≥ $3,000).

Marital status was categorized as never married, married, divorce or separated, and other or no response. Physical exercise was asked if the participants usually exercise enough to be sweat. Their nutritional intake (total calories [kcal/d], protein [g/d], fat [g/d], and carbohydrate [g/d]) was surveyed using a food-frequency questionnaire, which was validated in a previous study.^[14]^ They were also asked about past medical history of metabolic disease, hypertension, diabetes mellitus, and hyperlipidemia. Obesity was measured by BMI (kg/m2) using height and weight as continuous variables. Total smoking histories were calculated as pack-year, and alcohol consumption was measured as mean consumption of alcohol (g/d) using information about consumption frequency and types of alcohol consumed. In this study, we categorized FH of PCa into groups:

- total,
- father, and
- brother.

### 2.4 Statistical analyses

The Chi-squared test, or Fisher exact test was used to compare the rates of income, metabolic disease histories, and FH of PCa between PCa and control groups. The independent *t* test was used to compare age, BMI, smoking pack-year, and alcohol consumption. A stratified logistic regression model was used to analyze the odds ratio (OR) of PCa development for FH of PCa and to adjust possible bias. In the crude model, we only inserted each FH of PCa as an independent variable. In model 1, we inserted FH of PCa (father or brother) and age, income, BMI, smoking, alcohol intake, and medical history of hypertension, diabetes mellitus, and dyslipidemia as the independent variables. In model 2, we inserted PCa history of father, and PCa history of brother as independent variables. In model 3, we inserted the variables of model 2 and model 3. 95% confidence intervals (CI) were calculated. For the subgroup analysis, we divided the participants by age at registry (< 60 years old; ≥ 60 years old). Two-tailed analyses were conducted, and *P* values less than .05 were considered significant. The results were statistically analyzed using SPSS version 22.0 (IBM, Armonk, NY).

## 3 Results

Table 1 shows the differences in general characteristics between the PCa and control groups. The median age at registry participation of 69,693 men included in the analysis was 65 (range, 44–89) years and 100 (0.14%) men were PCa patients. Mean age of the PCa group was higher compared to that of the control group (65.3 ± 6.1 vs 54.8 ± 9.1 years, respectively, *P* < .001). In the PCa group, the percentage of patients with hypertension, diabetes, and dyslipidemia was higher than that in the control group. The mean g/d of alcohol consumption was higher in the control group (each of *P* < .05). Of 290 men identified with a positive PCa FH, 6 (2.1%) patients were diagnosed with PCa, whereas there were 94 (0.1%) PCa patients among 69,403 men without a FH of PCa. Among the entire cohort, a FH of father, or brother was identified in 240 and 50 cases, respectively. There were no cases with a history of PCa for both father and brother at the same time.

**Table 1:** General Characteristics of Participants.

	Total participants		
Characteristics	Prostate cancer (n, %)	Control (n, %)	*P* value
Total participants	100 (100.0)	69,593 (100.0)	
Age (yrs-old, mean, SD)	65.3 (6.1)	54.8 (9.1)	<.001^∗^
Income (n, %)			.19
No information	21 (21.0)	12,627 (18.1)	
Lowest	23 (23.0)	12,486 (17.9)	
Middle	29 (29.0)	18,796 (27.0)	
Highest	27 (27.0)	25,684 (36.9)	
Marital status (n, %)			.195
Never married	2 (2.0)	1,582 (2.3)	
Married	88 (88.0)	64,360 (92.5)	
Divorced or separated	7 (7.0)	2,398 (3.4)	
Other or no response	3 (3.0)	1,253 (1.8)	
Physical exercise (n, %)	65 (65.0)	36,454 (52.4)	.012^∗^
Nutritional intake (mean, SD)			
Total calories (kcal/d)	1720.9 (418.9)	1843.7 (573.2)	.032^∗^
Protein (g/d)	59.0 (22.2)	62.1 (26.9)	.250
Fat (g/d)	26.0 (15.1)	30.0 (19.0)	.033^∗^
Carbohydrate (g/d)	308.5 (86.0)	325.8 (91.9)	.061
Hypertension (n, %)	35 (35.0)	15,872 (22.8)	.01^∗^
Diabetes (n, %)	16 (16.0)	6,591 (9.5)	.03^∗^
Dyslipidemia (n, %)	18 (18.0)	6,228 (8.9)	.01^∗^
Obesity (BMI, kg/m^2^, mean, SD)	24.1 (2.8)	24.4 (2.8)	.39
Total smoking duration (pack-year, mean, SD)	20.0 (17.5)	17.9 (15.2)	.25
Drinking alcohol (g/day, mean, SD)	8.8 (17.8)	17.2 (33.8)	<.001^∗^
Family history			
Total	6 (6.0)	284 (0.4)	<.001^∗^
Father	4 (4.0)	236 (0.3)	<.001^∗^
Brother	2 (2.0)	48 (0.1)	<.001^∗^

Besides FH, age at registry participation (OR, 1.17; 95% CI, 1.14–1.21; *P* < .001), and hyperlipidemia (OR, 2.25; 95% CI, 1.32–3.84; *P* = .01) were identified as significant predictors of PCa diagnosis among the adjusted variables included in the multivariable logistic regression analysis. The adjusted ORs (model 3) of PCa history of father was 27.7 (95% CI = 9.7–79.2, *P* < .001) in the PCa group compared to the control group, and that of PCa history of brother was 15.8 (95% CI = 3.6–69.6, *P* < .001, Table 2), showing that the risk of developing PCa was greater in ascendant FH than fraternal FH. In the subgroup analyses according to age, adjusted ORs (model 3) of PCa history of father was 18.8 (95% CI = 2.3–150.7, *P* = .006) in the younger age group (Table 3). The adjusted ORs of PCa history of father was 35.6 (95% CI = 10.3–123.1, *P* < .001), and that of PCa history of brother was 15.4 (95% CI = 3.3–71.2, *P* < .001) in the older age group. These results also show that, regardless of age, the risk of developing prostate cancer was greater in ascendant FH than fraternal FH. In the analyses according to age of onset of prostate cancer, 1 patient did not have information on the onset age of PCa and was therefore excluded. There was no significant difference between the groups stratified by age of onset of PCa in terms of FH of PCa (Table 4, all *P* > .05).

**Table 2:** Association between prostate cancer history of the participants and their family histories of prostate cancer.

	ORs for prostate cancer							
	Crude		Model 1		Model 2		Model 3	
Each of history	OR (95% CI)	*P* value	OR (95% CI)	*P* value	OR (95% CI)	*P* value	OR (95% CI)	*P* value
Prostate cancer history, total								
Yes	15.6 (6.8–35.9)	<0.001^∗^	22.4 (9.4–53.4)	<.001^∗^	N/A		N/A	
No	1.00		1.00					
Prostate cancer history of father								
Yes	12.3 (4.5–33.6)	<0.001^∗^	27.2 (9.31–75.4)	<.001^∗^	12.5 (4.6–34.3)	<.001^∗^	27.7 (9.7–79.2)	<.001^∗^
No	1.00		1.00		1.00		1.00	
Prostate cancer history of brother								
Yes	29.6 (7.1–123.4)	<0.001^∗^	15.2 (3.40–66.8)	<.001^∗^	30.7 (7.4–128.2)	<.001^∗^	15.8 (3.6–69.6)	<.001^∗^
No	1.00		1.00		1.00		1.00	

**Table 3:** Subgroup analyses of association between prostate cancer diagnosis and family history of prostate cancer according to age.

	ORs for prostate cancer			
	Young (<60 yrs old, n = 46,445)		Old (≥60 yrs old, n = 23,248)	
Each of history	OR (95% CI)	*P* value	OR (95% CI)	*P* value
Prostate cancer history, total				
Yes	15.2 (1.9–121.4)	0.010^∗^	24.8 (9.3–66.3)	<.001^∗^
No	1.00		1.00	
Prostate cancer history of father				
Yes	18.8 (2.3–150.7)	0.006^∗^	35.6 (10.3–123.1)	<.001^∗^
No	1.00		1.00	
Prostate cancer history of brother				
Yes	No convergence		15.4 (3.3–71.2)	<.001^∗^
No	1.00		1.00	

**Table 4:** Ratio of family history of prostate cancer according to the onset of prostate cancer among prostate cancer participants.

	Onset of prostate cancer		
History	< 60 yrs old	≥ 60 yrs old	*P* value
Prostate cancer history, total (n, %)			
Yes	2 (7.4)	4 (5.6)	.66
No	25 (92.6)	68 (94.4)	
Prostate cancer history of father (n, %)			
Yes	2 (7.4)	2 (2.8)	.29
No	25 (92.6)	70 (97.2)	
Prostate cancer history of brother (n, %)			
Yes	0 (0.0)	2 (2.8)	.99
No	27 (100.0)	70 (97.2)	

## 4 Discussion

This study shows increased ORs of developing PCa according to type of FH (father vs brother) and different age groups (< 60 years vs ≥ 60 years old), ranging from 12.25 to 33.32. However, previous studies have reported that the heritability of PCa carries a 2.0 to 2.5-fold increased risk of developing PCa,^[6,15,16]^ which is significantly lower than the increased risk calculated by our study. This difference in OR values is thought to be due to the differences in study design and patient cohort. The population-based cohort used in our study consisted of community dwellers without any intend for participations rather than cohorts based on PSA screening, health examinations, or PCa patients, as was the case in the previous studies. We believe, therefore, that our analysis may have produced more natural results without any manipulation of the data regarding the impact of FH on the risk of developing PCa. In particular, the number of healthy controls was considerably higher than that of patients with PCa, so the OR values may have been different from the previous studies. Another possible explanation for the significantly higher OR values is likely related to PSA screening. The incidence of PCa has increased substantially since the introduction of PSA screening.^[17,18]^ However, the results from most previous studies are derived from data collected before or at the beginning of the PSA screening era. Due to PSA screening, a noticeable number of patients with PCa, who had not been detected before, may have been detected; therefore, the association of FH with the risk of developing PCa may have been prominently shown in our study, which did not appear previously. Indeed, hazard ratios from a large family study on the risk of developing PCa ranges from as low as 1.5 to 6.1, which is based on Swedish registry data collected between 1958 and 2006.^[8]^ On the contrary, the KoGES data used in our study was collected after 2001 when PSA screening had already been widely implemented in Korea and, therefore, probably led to significantly higher OR values. In addition, the fact that the number of PCa patients is significantly smaller than that of the control group may also have influenced the extreme values of OR from the statistics.

An FH is known to be associated with a higher potential risk of developing PCa at a younger age.^[15,19]^ In particular, European data reported that the risk of both PCa diagnosis and death increased with the number of affected family members, and this tendency was stronger in the younger age group.^[8]^ An Asian series with 602 patients similarly showed that patients with a FH were significantly younger.^[19]^ On the other hand, other researchers reporting the data of 257 men with PCa diagnosed at age 55 years or younger have shown that almost half of the patients reported a negative FH of PCa,^[20]^ indicating that there is no association between the early onset PCa and a FH of the disease. Our result showed that the mean age of PCa patients with a FH was younger than those without a FH, although it was not statistically significant (58.3 ± 3.9 vs 62.4 ± 6.4 year, respectively, *P* = .142). However, as Table 4 shows, our analysis failed to meet the statistical significance in the association between the age of onset of PCa and the number of patients with a FH among PCa patients, suggesting that the significance of FH in the age of onset of PCa needs to be further investigated.

of the advantages of our study is that we analyzed FH by separating those of fathers and brothers, and also by combining them. Although the majority of previous studies analyzed FH without separating them, a study of Swedish registry data showed different risks according to patient age and type of FH (father vs brother).^[8]^ In this study, the risk of PCa diagnosis in patients with a FH of brother was consistently higher than those in patients with a FH of father. Additionally, a meta-analysis showed that PCa patients with an affected brother showed a higher risk than those with an affected father (relative risk, 2.87 vs 2.12, respectively).^[16]^ However, in our analysis, the risk of PCa development was higher in patients with a FH of father than in those with a FH of brother, as shown in Table 2 (ORs, 27.7 vs 15.8, respectively), which was quite the opposite of previous results. The result was consistent in the sub-analysis according to patients’ age at registry participation (Table 3). This inconsistency between previous results and ours may be due to a notable difference in the patient cohort. The abovementioned Swedish data originated from a registry database of a cancer population between 1961 and 2006. In contrast, our results were derived from those of healthy community dwellers, regardless of cancer diagnosis, between 2001 and 2013, indicating that our patient cohort consists of a more generalized population and recent data.

Another interesting finding from the current analysis that differs from the previous study is that the proportion of patients with a FH of PCa is low in PCa patients. Western data shows that the proportion of patients with PCa who have a FH of PCa ranges approximately from 5.4% to 21%.^[8,16,21]^ Except for 1 study, which was conducted in 1996,^[22]^ the proportion was over 10% in most Western data. In particular, a large twin study reported that heritable factors may have been attributable to up to 42% of PCa cases,^[6]^ which was much higher than our result of 6% (6/100). This discrepancy, it can be assumed, was caused by ethnic differences. The fact that the rates or tumor biology of PCa vary substantially by race, ethnicity, and geography is widely known from previous studies.^[23,24]^ It can also be suggested that the relatively low incidence of PCa in an Asian population may have resulted in a lower proportion of patients with a FH compared to previous Western data. Indeed, a recent study from a Korean series also showed a relatively low proportion of FH, reporting 6.8% had a FH in PCa patients.^[19]^ Therefore, to summarize, our results were consistent with previous Western series that FH is associated with an increased risk of developing PCa. However, a few important differences were noted in the present study: a FH of father had a greater impact on the increased risk of developing PCa than a FH of brother; and, overall, the proportion of patients with a FH was low in PCa patients, while the impact of FH on the increased risk of developing PCa was much higher than in Western data.

Although our study is the first, to our knowledge, to report the significance of FH in PCa development among Korean population using a registry database, it had several limitations. First, it was impossible to include the effect of age of onset of PCa in family members due to the lack of recorded data. Second, it may be possible that men with a FH of PCa are more likely to be exposed to PCa screening than those who do not have a FH, and thus have a higher rate of PCa diagnosis, rather than a FH itself having an impact on PCa development. Accordingly, a report of Finish data demonstrated the risk for PCa diagnosis associated with a positive FH did not substantially increase among patients who underwent PSA screening and subsequent prostate biopsy.^[25]^ Therefore, further investigation is warranted to elucidate the association between FH and PSA screening. Third, since the pathologic findings are not included in the KoGES data, we were unable to assess whether FH was associated with pathologic outcomes of PCa. Although previous studies reported that FH in PCa patients were associated with low-risk disease or better survival outcome,^[26,27]^ this also needs to be validated in the Korean population. And lastly, the sample size of PCa patients was small; the number in the patient group was only 0.1% of the number in the control group (100 vs 69,593). If the number in the PCa group were greater, we believe that the reliability of statistical power would be higher. Since the prevalence of cancer disease is very low compared to diseases such as diabetes and hypertension, despite the investigation of considerably large number of subjects, the proportion of PCa patients is very low, and there is disadvantage that subjects were not secured sufficiently. In order to compensate for such shortcomings, a complementary follow-up study with more than 100,000 subjects must be followed. Nevertheless, presenting these contemporary data has its own significance as a preliminary result on FH and PCa in Korean population.

## 5 Conclusion

The risk of developing PCa is significantly increased in men with an overall FH of PCa in a Korean general population. However, the association between FH of PCa and early onset of the disease does not appear to be clear and this needs to be investigated further. These specific findings should guide clinical counseling for Korean men in recommendations for screening.

## Acknowledgments

The authors would like to thank the highly qualified native English-speaking editors at American Journal Experts (Enago, the editing brand of Crimson Interactive Korea & Co. Ltd.) for proper English language, grammar, punctuation, spelling, and overall style.

## Author contributions

**Conceptualization:** Hyo Geun Choi, Myungsun Shim.

**Data curation:** Hyo Geun Choi, Woo Jin Bang, Jung Ki Jo, Cheol Young Oh, Jin Seon Cho, Myungsun Shim.

**Formal analysis:** Hyo Geun Choi, Myungsun Shim.

**Funding acquisition:** Hyo Geun Choi.

**Investigation:** Myungsun Shim.

**Methodology:** Hyo Geun Choi, Myungsun Shim.

**Project administration:** Hyo Geun Choi.

**Resources:** Hyo Geun Choi.

**Software:** Hyo Geun Choi.

**Supervision:** Myungsun Shim.

**Writing – original draft:** Hyo Geun Choi, Myungsun Shim.

**Writing – review & editing:** Myungsun Shim.
